# Enamel Defects of Human Primary Dentition as Virtual Memory of Early Developmental Events

**DOI:** 10.5681/joddd.2009.028

**Published:** 2009-12-15

**Authors:** Naser Asl Aminabadi, Sina Ghertasi Oskouei, Firoz Pouralibaba, Zahra Jamali, Farzaneh Pakdel

**Affiliations:** ^1^ Associate Professor, Department of Pediatric Dentistry, Faculty of Dentistry, Tabriz University of Medical Sciences, Tabriz, Iran; ^2^ Research Assistant, Faculty of Dentistry, Tabriz University of Medical Sciences, Tabriz, Iran; ^3^ Assistant Professor, Department of Oral Medicine, Faculty of Dentistry, Tabriz University of Medical Sciences, Tabriz, Iran; ^4^ Post-graduate Student, Department of Oral Medicine, Faculty of Dentistry, Tabriz University of Medical Sciences, Tabriz, Iran

**Keywords:** Developmental, enamel defects, primary dentition

## Abstract

**Background and aims:**

The objectives of the present study were to investigate the prevalence and the position of enamel defects of primary teeth and hence to estimate the approximate time of an insult.

**Materials and methods:**

121 children aged 3 to 5 years were included in the study. The Modified Developmental De-fects of Enamel Index was used to diagnose and classify the defects. The defects were categorized as hypoplasia, hypocalcification or a combination of them. Each tooth was investigated for occlusal/incisal, middle, cervical, incisomiddle, cervicomiddle and complete crown defects.

**Results:**

55.37% of the children were affected by enamel defects, 23.96% being categorized as hypocalcification and 22.31% as hypoplasia. The enamel defects were more abundant in maxillary primary incisors and mandibular primary canines. Minimum involvement was seen in maxillary primary second molars and mandibular primary lateral incisors. The prevalence of cervical defects in maxillary primary incisors was significantly more than the middle or incisal defects (P < 0.05). The prevalence of incisal defects in mandibular primary incisors was significantly more than the middle or cervical defects (P < 0.05).

**Conclusion:**

The results revealed a considerable number of enamel defects which are multiple, symmetric and chrono-logically accordant with the estimated neonatal line in primary teeth of healthy children.

## Introduction

Human primary teeth develop during pregnancy and early childhood.^1 , 2^ Because the developing tooth germ is sensitive to a wide range of systemic disturbances and is unable to recover once it is damaged, the tooth enamel often acts as a repository of information on systemic insults received during development. Enamel defects of the primary teeth may have the potential for identification of children who have undergone certain systemic insults during prenatal and early postnatal life and also for estimating the timing of these events on a scale of days and weeks rather than months and years.^3 , 4^

The neonatal ring is an important landmark in establishing the chronology of deciduous enamel formation and helps distinguish prenatally formed enamel from postnatal enamel.^5^ An accepted standard chart, for the chronology of tooth development,^6^ was offered by Lunt and Law^1^ revising the one offered by Massler et al after a careful review of literature on the calcification of the primary teeth. The age at which a defect formed, or the ontogenetic timing of the stress episode, can be estimated from its position on the tooth crown according to this chart.^3^

All enamel defects are considered as indications of severe stress regardless of its duration, as they result from a cellular disruption during development,^7 , 8^ which may occur in the histodifferentiation, apposition, or mineralization stages of tooth development.^9^ Ameloblasts are extremely sensitive, and if disturbed during their secretory phase, a reduced thickness of normal enamel or a quantitative defect occurs and is regarded as hypoplasia. However, as for hypomineralization or opacities, or qualitative defects, the ameloblasts must be affected in the later mineralization or maturation phase of amelogenesis.^10^

On primary teeth, it may be possible to estimate the approximate timing of an insult relative to the position of the defect using the estimated neonatal line as a reference marker.^11^ While this indicator has been widely used in anthropological studies among skeletal populations, it is surprising that the validity of this estimated indicator has not been well tested among living populations. The aims of this study were to investigate the prevalence and the position of hypoplastic and/or hypocalcified enamel defects and hence to estimate the approximate time of an insult. It was hypothesized that the chronologic distribution of these defects would be a reliable marker to determine the chronology of many systemic insults recorded in deciduous tooth enamel.

## Materials and methods

The present study has two main components. The first component is an empirical analysis to investigate the prevalence of hypoplastic and/or hypocalcified enamel defects. The second component is a theoretical comparison of the position of these defects on tooth crowns by use of the estimated neonatal line as a marker. This component is aimed at identifying the estimated ages of defect formation.

The children invited to participate in the study were referrals from pediatric hospital where they were under routine care. All the children resided in regions with less than 0.32 ppm fluoride in their drinking water. The subjects were selected through simple random sampling and careful screening for inclusion criteria which allowed:

Maternal factors:

- Maternal age: 18–35 yr
- No preterm deliveries or perinatal death
- No drug or alcohol abuse and no cigarette consumption
- No serious maternal illness

Infant factors:

- Gestational age 38–41 wk
- Birth weight > 2500 g
- Apgar score at 1 min > 6; at 5 min > 7
- No resuscitation procedures performed
- No sign of illness before the examination

A case history of each child’s first 2 yr of life was then taken, including:

- Residence in Tabriz
- No history of systemic or debilitating diseases or local confounding factors, e.g. traumatic habits.

The participants included 121 children aged 3 to 5 years, of both genders, enrolled in the Department of Pediatric Dentistry, Tabriz University of Medical Sciences in 2009. Sample size was restricted by the number of healthy children meeting inclusion criteria for study.

The study design was submitted to and approved by the Committee for Ethics in Research on Humans, Tabriz University of Medical Sciences. A structured questionnaire was prepared and involved maternal factors, patient information, prenatal, perinatal, and postnatal problems, Apgar index, and enamel hypoplasia and hypocalcification. The parents of the children completed the questionnaire about the use of medication, fluoride, inclusion and exclusion factors pertaining to them. Data from hospital records were obtained where necessary.

The Modified Developmental Defects of Enamel Index (Modified DDE Index)^12^ was used to diagnose and classify changes in the enamel of the deciduous teeth studied. Three surfaces were examined in all deciduous teeth: buccal, occlusal/incisal and lingual/palatal. The enamel defects were assessed according to three clinical aspects as follows:^1 , 1^

- Demarcated opacity: Defect involving an alteration in the translucency of the enamel, variable in degree. The defective enamel is of normal thickness with a smooth surface. It has a distinct and clear boundary with the adjacent normal enamel and can be white, cream, yellow or brown in color
- Diffuse opacity: Defect involving an alteration in the translucency of the enamel, variable in degree and white in color. The defective enamel is of normal thickness and can have a linear, patchy or confluent distribution, but there is no clear boundary with the adjacent normal enamel
- Hypoplasia: Defect involving the surface of the enamel associated with a reduced localized thickness of enamel without dentinal exposure or complete absence of enamel over a considerable area of dentine. It may occur in the form of pits, grooves, or larger areas of missing enamel.

Both opacity and hypoplasia were recorded when defect involved the surface of the enamel with an alteration in its translucency, diffuse or demarcated, associated with partial or complete absence of enamel over a considerable area of dentine. Other diagnostic criteria were considered, namely, a) a tooth was considered present when any portion of the crown had erupted through the mucosa; b) when an enamel defect was present in the erupted portion, it was recorded; c) in the case of doubt regarding the presence of an abnormality, the dental surface was classified as “normal”; d) a surface with a single abnormality less than 1 mm in diameter was classified as “normal”; e) the dental surfaces that presented marked fractures, caries and very extensive restorations, or impacting on more than two thirds of the tooth surface were excluded from the analysis and classified as “excluded” and f) all of the deciduous teeth extracted or exfoliated were considered “excluded.” ^15^

**Figure 1.**
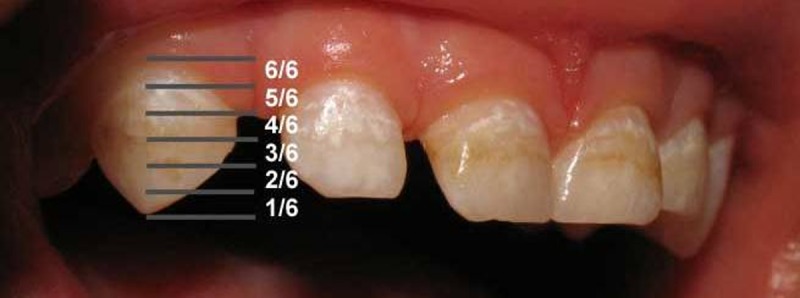
Six divisions used to determine the position of defects on the tooth crown according to the table of Lunt & Law.

For determining the position of the enamel defect on the tooth crown, teeth were divided to six portions: Occlusal/incisal, middle, and cervical one third; incisomiddle and cervicomiddle two thirds, and complete crown defects. Position of the estimated neonatal line was acquired from the table of Lunt & Law.^6^ Each one third was further divided into two subdivisions to estimate the time of an insult using the data from the table of Lunt & Law (Figure 1). Those enamel defects were identified as parts of the neonatal line that were correspondent to the estimated neonatal line.

Dental examinations were made by an experienced pedodontist, assisted by one trained dentist. The examinations were carried out under artificial illumination on a dental chair. For improved visualization, children were examined after dental prophylaxis and drying of their teeth. Flat oral mirrors and sharp dental explorers were used for the examination. If any tooth surface had a detectable softened area, the lesion was diagnosed as caries. Furthermore, the child was classified as having early childhood caries (ECC) when presenting with one or more decayed, missing, or filled tooth surfaces. Children were classified as presenting severe ECC when dental caries was present on any smooth surface. White spot lesions were not scored as carious lesions and were differentiated from enamel opacities when they were located adjacent to the gingival margin, and extended along the buccal or lingual surfaces. In contrast, enamel opacities have no preferential location on the tooth.^1 , 16^

To evaluate the intra-examiner reproducibility of the investigator, on a random basis, 25 subjects were re-evaluated after 1 week by the same blinded examiner to assess intra-examiner agreement of data.

The prevalence of defects in the enamel was calculated with a respective confidence interval of 95% and the distribution of the defects by arch, teeth and type of defect. Intra-examiner agreement of data for examinations was evaluated by Cohen’s kappa statistics. Statistical significance for differences between proportions was assessed using the chi-squared test while numerical variables were tested using Mann-Whitney U-test and Kruskal-Wallis H-test. The level of significance was set at 5%.

## Results

A total of 121 children aged 3 to 5 years were included in the present study. The Kappa value for intra-examiner agreement was 0.91. This demonstrates almost high intra-examiner agreement (P < 0.05).

### Maxillary teeth

The distribution pattern of enamel defects in primary maxillary teeth is presented in Table 1. These defects were more abundant in the cervical third of the maxillary primary incisors (67% of A, 61% of B). However, there were no cervical defects in primary maxillary canine and molars. These teeth were more frequently affected by incisal/occlusal defects. In addition, most of the enamel aberrations were found on the occlusal third of primary maxillary molars (83.67% of D, 83.87% of E).

**Table 1:** Site-specific distribution of enamel defects in maxillary teeth

	Incisal/occlusal third		Middle third		Cervical third		Incisal and middle two-thirds		Cervical and middle two-thirds		Complete crown defect		Total	
Teeth	n	%	n	%	n	%	n	%	n	%	n	%	n	%
A	6	5.9	10	9.8	68	66.7	1	0.98	5	4.9	12	11.8	102	22.3
B	6	6.5	5	5.4	56	60.9	9	9.8	7	7.6	9	9.8	92	20.8
C	36	94.7	2	5.3	0	0	0	0	0	0	0	0	34	7.4
D	41	83.67	1	2	0	0	4	8.33	0	0	3	6.25	49	10.69
E	26	83.87	1	3.2	0	0	2	6.66	0	0	2	6.66	31	6.7

The presence of enamel defects was a more common finding in primary maxillary incisors (> 20%). Minimum involvement of maxillary primary teeth by enamel defects was observed for primary second molars (6.7%) followed by primary canine (7.4%) and primary first molar (10.69%).

###  Mandibular teeth

The distribution pattern of the enamel defects in primary maxillary teeth is presented in Table 2. These defects were more abundant in the middle third of the mandibular primary incisors (61.5% of A, 47.8% of B). Interestingly, the distribution of enamel defects in the mandibular posterior teeth followed a pattern similar to that of maxillary teeth. There were no cervical defects in primary maxillary canines. These teeth were more frequently affected by incisal/occlusal defects. The defects of the cervical and middle third did not exist in mandibular primary molars. These teeth exhibited occlusal defects in most of the cases.

**Table 2:** Site-specific distribution of enamel defects in mandibular teeth

	Incisal/occlusal third		Middle third		Cervical third		Incisal and middle two-thirds		Cervical and middle two-thirds		Complete crown defect		Total	
Teeth	n	%	n	%	n	%	n	%	n	%	n	%	n	%
A	0	0	16	61.5	10	38.5	0	0	0	0	0	0	26	5.67
B	0	0	11	47.8	12	52.2	0	0	0	0	0	0	23	5.02
C	32	94.1	1	2.9	0	0	0	0	0	0	1	2.9	42	9.1
D	30	85.7	0	0	0	0	3	8.57	0	0	2	5.7	35	7.64
E	20	100	0	0	0	0	2	8.33	0	0	2	8.33	24	5.24

The presence of enamel defects was a more common finding in primary mandibular canines (9.1%). Minimum involvement of mandibular primary teeth by enamel defects was observed for primary lateral incisor (5.02%) followed by primary second molar (5.24%), primary central incisor (5.67%), and primary first molar (7.64%).

###  Defect types

Table 3 shows the distribution of hypoplasia and hypocalcification in the studied subjects. 55.37% of the children were affected by some type of enamel defects. Of these, 23.96% were categorized as hypocalcification and 22.31% were hypoplasia. On a teeth-based scale, enamel defects were detected on 22.23% of the teeth. Hypocalcified areas were detected in 11.11% of the teeth, and 9.31% of the teeth were affected by hypoplasia.

**Table 3:** Distribution of enamel defects according to defect types

	Children		Teeth	
	n	%	n	%
Hypocalcification (opacity)	29	23.96	228	11.11
Hypoplasia	27	22.31	191	9.31
Hypoplasia + Hypocalcification	11	9.09	39	1.9
Total Defects	67	55.37	458	22.23
Total	121	100	2051	100

### Bivariate analysis

The prevalence of cervical defects in maxillary primary incisors was significantly more than the middle or incisal defects (P < 0.05). However, there was no significant statistical difference in prevalence of middle and incisal defects (P > 0.05).

The prevalence of incisal defects in mandibular primary incisors was significantly more than the middle or cervical defects (P < 0.05). However, there was no significant statistical difference in prevalence of middle and cervical defects (P > 0.05).

While there was no significant difference between the prevalence of hypocalcified and hypoplastic defects (P > 0.05), the prevalence of combined hypoplastic/hypocalcified defects was significantly less than individual types in both maxilla and mandible (P < 0.05).

## Discussion

In accordance with other studies,^15 , 17^ among the three types of defects examined in our study, diffuse opacities were the most commonly found defect in children (23.96%). The teeth most commonly affected by defects among maxillary teeth were the central incisors, while the canines were the most affected ones among mandibular teeth. As a whole, the teeth most affected by defects were the incisors. This finding coincides with that of other studies in which the incisors were the most affected teeth.^18 , 19^ In contrast, there are studies which disagree with our findings, indicating that molars were the most affected.^15 , 20 , 21^ When analysis of the distribution of enamel defects is focused on which tooth segments are affected, available data reflect differences in the studied samples. It is interesting to note that the most of enamel aberrations distributed on the gingival half of the primary incisors, middle third of primary canines and on the occlusal third of primary molars in both jaws. This finding is in accordance with the chronology of tooth development,^6^ and can be explained by the fact that although there is considerable variation in children in terms of relative timing of enamel calcification of individual teeth, molars have probably completed a little more than the cusp formation at the time that hypoplasia develops in the anterior teeth.^22^

**Figure 2:**
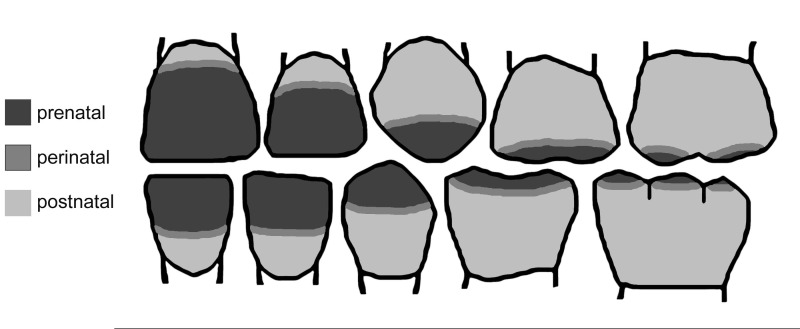
Multiple, symmetric enamel defects were more prevalent than those of the other segments on the tooth crown. These were chronologically accordant with the neonatal line.

It would be of interest further to note that enamel defects which are multiple, symmetric and chronologically accordant with the estimated position of neonatal line were more prevalent than those of the other segments on the tooth crown (Figure 2). As the major part of the enamel defects in the primary dentition was symmetrical, it is reasonable to speculate that common systemic etiological factors are responsible for enamel defects in the primary teeth.

This can also be attributed to the wider neonatal line, and therefore, it can be regarded as sign of neonatal situational stress. At birth, the infant is suddenly separated from maternal nutrition and must establish its own system of diurnal rhythms.^2^

One of several proposed causative factors of enamel aberrations is hypocalcemia. The clinical evidence is strongly suggestive of disturbances in calcium homeostasis as one of the main pathways underlying enamel defects in primary teeth associated with systemic disturbances.^3 , 2 - 29^

The formation of enamel involves a rhythmic sequence of cellular activity interspersed with resting phases. Enamel, having very high calcium content, requires large numbers of calcium ions for mineralization. It is probable that a shortage of calcium ions severely disturbs the ameloblasts in certain developmental stages of the enamel.^29^

Selective involvement of only those ameloblasts that were currently active at the time of a particular disturbance may account for the variability in development of the lesions. Some of the affected ameloblasts may die and stop secreting enamel, whereas others may recover and continue to secrete normal enamel over the defective spots, which could also help to explain the variability of the enamel lesions.^3 , 3^

Neonatal changes of calcium metabolism belong to the group of general factors which might disturb amelogenesis, and thus, all teeth undergoing mineralization at the time could be affected.^29^ The results show that measured low values of ionized blood calcium on the first days after birth cannot alone explain the neonatal line width. Among other theories of why the neonatal line occurs, the trauma implicit in birth has been proposed as a factor of importance.^26 , 31^

Systemic disturbances occurring neonatally in a full-term infant with normal dental development should, according to the literature, give rise to enamel aberrations distributed on the gingival half of the primary incisors, and incisally on primary canines and on the occlusal half of primary molars.^1^

Herman and McDonald found a definite relationship between the time of possible factors that could have caused brain damage and the apparent time of the enamel defect based on its position in the enamel on the crown of the tooth.^6^ So, the evidence of enamel hypoplasia is an aid to the clinicians and the researchers in determining when brain injury occurred in the patients in whom the cause is not clearly defined. Cohen and Diner observed that chronologically distributed enamel defects were a valuable aid in neurologic diagnosis, since they occur commonly in brain-damaged children. In addition, the defects indicate the time of insult to the developing fetus or infant even when the history is reportedly negative.^6^

In this study, however, enamel defects were observed on the incisal/occlusal and middle as well as complete crown surface of primary incisors and molars. Considering the chronology of the development and calcification of the deciduous teeth and the estimated position of the neonatal line as a marker, it is possible to conclude that many unclear factors in addition to systemic insults on the first few days of life in healthy neonates are responsible for the results of our study.

Although primary teeth are important as they present the only form of observable records from prenatal and perinatal events of human life, we regard these types of data not as standards, but rather as illustrative of several key facts that are still not well-understood or which are still ignored. It is apparent that many questions remain regarding factors affecting enamel formation during the prenatal, perinatal and postnatal periods of healthy neonates. The present study was not directed at the detection of the pathogenesis of enamel hypoplasia but rather to its position on tooth crown. Further studies will be needed in order to assess possible mechanisms involved in these defects.

Preliminary results of the present study revealed a considerable number of enamel defects in primary teeth of healthy children which were mainly chronologically accordant with the neonatal line. It is surprising that this indicator has not been well tested among living populations. More research on other populations towards the prevalence and timing of these defects using neonatal line as indicator is suggested.
